# Assessment of iron status in settings of inflammation: challenges and potential approaches

**DOI:** 10.3945/ajcn.117.155937

**Published:** 2017-10-25

**Authors:** Parminder S Suchdev, Anne M Williams, Zuguo Mei, Rafael Flores-Ayala, Sant-Rayn Pasricha, Lisa M Rogers, Sorrel ML Namaste

**Affiliations:** 1 Department of Pediatrics, Emory University, Atlanta, GA;; 2 Nutrition Branch, CDC, Atlanta, GA;; 3 Medical Research Council (MRC) Human Immunology Unit, MRC Weatherall Institute of Molecular Medicine, University of Oxford, Oxford, United Kingdom;; 4 Department of Nutrition for Health and Development, WHO, Geneva, Switzerland; and; 5 Helen Keller International and Strengthening Partnerships, Results, and Innovations in Nutrition Globally, Arlington, VA

**Keywords:** infection, inflammation, iron status, preschool children, serum ferritin, soluble transferrin receptor, total-body iron stores, women of reproductive age

## Abstract

The determination of iron status is challenging when concomitant infection and inflammation are present because of confounding effects of the acute-phase response on the interpretation of most iron indicators. This review summarizes the effects of inflammation on indicators of iron status and assesses the impact of a regression analysis to adjust for inflammation on estimates of iron deficiency (ID) in low– and high–infection-burden settings. We overviewed cross-sectional data from 16 surveys for preschool children (PSC) (*n* = 29,765) and from 10 surveys for nonpregnant women of reproductive age (WRA) (*n* = 25,731) from the Biomarkers Reflecting the Inflammation and Nutritional Determinants of Anemia (BRINDA) project. Effects of C-reactive protein (CRP) and α1-acid glycoprotein (AGP) concentrations on estimates of ID according to serum ferritin (SF) (used generically to include plasma ferritin), soluble transferrin receptor (sTfR), and total body iron (TBI) were summarized in relation to infection burden (in the United States compared with other countries) and population group (PSC compared with WRA). Effects of the concentrations of CRP and AGP on SF, sTfR, and TBI were generally linear, especially in PSC. Overall, regression correction changed the estimated prevalence of ID in PSC by a median of +25 percentage points (pps) when SF concentrations were used, by −15 pps when sTfR concentrations were used, and by +14 pps when TBI was used; the estimated prevalence of ID in WRA changed by a median of +8 pps when SF concentrations were used, by −10 pps when sTfR concentrations were used, and by +3 pps when TBI was used. In the United States, inflammation correction was done only for CRP concentrations because AGP concentrations were not measured; regression correction for CRP concentrations increased the estimated prevalence of ID when SF concentrations were used by 3 pps in PSC and by 7 pps in WRA. The correction of iron-status indicators for inflammation with the use of regression correction appears to substantially change estimates of ID prevalence in low– and high–infection-burden countries. More research is needed to determine the validity of inflammation-corrected estimates, their dependence on the etiology of inflammation, and their applicability to individual iron-status assessment in clinical settings.

## INTRODUCTION

Despite the negative health consequences of iron deficiency (ID), the magnitude and distribution of ID are largely unknown. The hemoglobin concentration that is used to define anemia is commonly assessed and used as a proxy for iron status, but this indicator is neither a sensitive nor specific measure of ID (1). For example, the WHO Micronutrients Database in the Vitamin and Mineral Nutrition Information System, which monitors data on indicators of vitamin and mineral nutrition in populations, has thus far included hemoglobin concentrations but not iron-status indicators (2). In the United States, iron status is routinely assessed through the NHANES. The Healthy People 2020 objectives, which aim in part to reduce ID in young children and women of childbearing age, have adopted the use of total body iron (TBI), the log ratio of soluble transferrin receptor (sTfR) to serum ferritin (SF) (used generically to include plasma ferritin) concentrations, to assess population-level iron status (3). The use and complexities of these indexes have been addressed elsewhere (4, 5) and in these proceedings (6).

## CONFOUNDING EFFECTS OF INFLAMMATION ON IRON INDICATORS

The interpretation of iron indicators is hindered by physiologic factors that can affect their concentrations and, in some cases, can result in failure to detect an iron-deficient state (7). In particular, inflammation, which is characterized by the acute-phase response (APR) to infection, injury, or environmental insults, can directly affect the concentrations of most iron indicators (8). The effects of the APR are typically temporary and self-limiting (8). SF is a positive acute-phase (AP) protein, which is markedly elevated during states of inflammation, most likely in response to increasing amounts of cytokines (9). Although sTfR appears to be less influenced by inflammation than SF is, sTfR concentrations increase in individuals with general inflammation (10), increased erythropoiesis from malaria infection (11), or red blood cell disorders (12). The interpretation of TBI is limited by the same confounding factors as for SF and sTfR concentrations because TBI is a combination of these 2 indicators (5).

The biological mechanisms that underpin the effect of inflammation on iron-status indicators are not clearly understood. As reviewed by Ross (13), both acute inflammation that is due to infection or injury and chronic inflammation, which results from metabolic disturbances, can affect iron trafficking in part through their effects on the regulation and synthesis of hepatic AP proteins. For example, the AP proteins ferritin, transferrin, haptoglobin, and hepcidin are induced by the APR and may affect the distribution of iron to cells throughout the body (13). Inflammation may also impair iron status by decreasing intake of food and reducing intestinal absorption (8, 14). Note that the interactions between inflammation and nutrition are bidirectional such that malnutrition itself can directly impact immune function and the APR (8).

The confounding effects of inflammation can result in an incorrect diagnosis of malnutrition in individuals as well as the overestimation or underestimation of the prevalence of deficiency in a population. For example, the elevation in SF concentrations with inflammation masks ID, which would normally be reflected by decreased SF concentrations. To date, there are no universally accepted approaches to account for inflammation in estimating micronutrient status. To assess population iron status with the use of SF concentrations, the CDC and WHO currently recommend conducting surveys in a low-inflammation season or measuring inflammatory indicators and either excluding individuals from the analysis who are inflamed or raising the SF cutoff to define deficiency (15, 16). The most commonly measured AP proteins in nutrition surveys are C-reactive protein (CRP), which is a measure of acute inflammation, and α1-acid glycoprotein (AGP), which is a measure of chronic inflammation (9). These and other inflammatory biomarkers that are used in research and clinical settings have been reviewed by other authors (8). New approaches to interpret iron indicators with the use of biomarkers of inflammation are emerging. An understanding of the relation between inflammation and nutrient biomarkers has been identified as a critical research gap by the National Institute of Child Health and Human Development Biomarkers of Nutrition for Development and the Inflammation and Nutrition Science for Programs and Interpretation of Research Evidence projects (8, 17).

Most studies that have examined the relation between iron-status indicators and inflammation have been conducted in low-income countries with high infection burden (18, 19); in contrast, there has been limited exploration of the importance of inflammation on estimates of iron status in high-income countries with low infection burden (7, 20). Iron-status data from the NHANES have shown that 14% of toddlers aged 1–2 y (21) and 18% of pregnant women (30% of whom are in the third trimester) are iron deficient as defined by TBI <0 mg/kg (22). However, these data were not adjusted for inflammation, and thus, the extent of ID in the United States may be higher than currently estimated (7, 20, 23).

## COMPARING APPROACHES TO ADJUST FOR INFLAMMATION

To address the challenges in the assessment of nutrition status, a collaborative research group called Biomarkers Reflecting Inflammation and Nutrition Determinants of Anemia (BRINDA) was formed in 2012 to analyze pooled data from population-based nutrition surveys and answer priority research questions that are related to the assessment of micronutrient status in settings of inflammation (24). With the use of select findings from 6 previously published reports from the BRINDA project (25–30), this review summarizes the magnitude of the effect of AP proteins (specifically CRP and AGP) on iron-status indicators (SF and sTfR concentrations and TBI), and assesses the potential impact of the use of regression to adjust for inflammation on estimates of ID in both low– and high–infection-burden settings. This review was intended to support discussions that were held as part of the workshop that forms the basis for these proceedings. In addition, we identify gaps in research that is related to the validation of iron assessment in settings of inflammation.

The methods of the BRINDA project have previously been published (30). In brief, the BRINDA project included national or regionally representative nutrition surveys that met the following inclusion criteria: *1*) surveys were conducted after 2004; *2*) target groups included preschool children (PSC) aged 6–59 mo, nonpregnant women of reproductive age (WRA) aged 15–49 y, or both groups; and *3*) surveys measured ≥1 indicator of iron status (SF or sTfR concentrations) and ≥1 biomarker of inflammation (AGP or CRP) (24). A total of 16 PSC and 10 WRA data sets were included for the analyses that are summarized in this paper (30). Because of the interest in the United States population, we compared results by the NHANES with other data sets. With the use of an approach that examined country-level characteristics to categorize countries into groups reflecting risk and burden of infectious disease (31), BRINDA countries were classified as low infection burden (United States and Republic of Georgia), medium infection burden (Colombia and Mexico), and high and very high infection burden (Cameroon, Cote d’Ivoire, Kenya, Liberia, Bangladesh, Laos, Papua New Guinea, and Philippines).

### Case definitions and statistical analysis

The 3 iron indicators that were evaluated in the BRINDA data sets were SF and sTfR concentrations and TBI. TBI was calculated from sTfR and SF concentrations with the use of the following formula from Cook et al. (32):


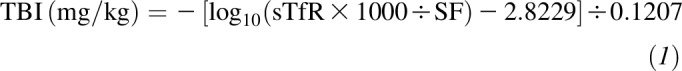


The following cutoffs were used to define estimated ID: SF concentration <12 μg/L in PSC and <15 μg/L in WRA; sTfR concentration >8.3 mg/L with the use of Ramco equivalent values in both PSC and WRA; and TBI <0 mg/kg in both PSC and WRA (4). Inflammation was defined as a CRP concentration >5 mg/L or AGP concentration >1 g/L (33). A summary of the laboratory methodology and quality control for each indicator as well as the statistical analysis approach have previously been published (30).

The following 3 primary approaches to adjust SF and sTfR concentrations and TBI for inflammation were explored (**Table 1**): *1*) the exclusion approach whereby observations were excluded that had elevated CRP or AGP concentrations, and the prevalence of estimated ID was calculated in the remaining individuals; *2*) a correction-factor approach (33) whereby arithmetic correction factors were calculated with the use of a 4-group inflammation-adjustment model of a reference (both CRP and AGP were normal), incubation (only CRP was elevated), early convalescence (both CRP and AGP were elevated), and late convalescence (only AGP was elevated) on the basis of each individual survey; and *3*) a regression-correction approach whereby linear regression was used to adjust the SF concentration by the concentrations of CRP and AGP and the sTfR concentration by the concentration of AGP on a continuous scale on the basis of each individual survey. Subsequently, inflammation-adjusted TBI were calculated with the use of adjusted sTfR and SF concentrations. Additional details of the inflammation-adjustment approaches, including the sample code, have previously been reported by the BRINDA working group and also summarized on the BRINDA website (www.BRINDA-nutrition.org) (30).

**TABLE 1:** Approaches to adjust iron indicators for inflammation: the BRINDA project^1^

Approach	Method
Unadjusted	No adjustments for AGP, CRP, or both.
Exclusion	Exclude individuals from data set with CRP concentrations >5 mg/L, AGP concentrations >1 g/L, or both.
	Calculate estimated prevalence of micronutrient deficiency with the use of remaining subsample.
CF	Stratify data set into groups by inflammation status depending on data availability and MB^2^
	Calculate the CF (ratio of the MB values’ GM in the reference group to the respective inflammation group) for each categorization with the formula shown in Equation *2*.
	Multiply the raw MB values by the appropriate group CF: 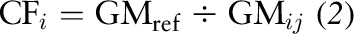 where *i* is the data set, *j* is the group, and ref is the reference group.
RC	Run linear regression models. The outcome variable is the ln MB. Depending on available data, ln CRP and ln AGP (continuous) can be included in the model as explanatory variables.Extract slopes from explanatory variables and input into the RC formula shown in Equation *3* (slope values are multiplied by CRP and AGP observations and subtracted from the MB observations).Back transform adjusted MB values before applying MB cutoffs. 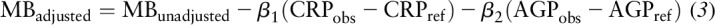 where β_1_ is the CRP RC, β_2_ is the AGP RC, obs is the observed value, and ref is the reference value. MBs CRP, AGP, CRP_ref_, and AGP_ref_ are on the ln scale. Reference values are the maximum value of the lowest CRP or AGP decile obtained from the combined BRINDA database. The unlogged reference values are as follows: CRP in PSC = 0.10, CRP in WRA = 0.16, AGP in PSC = 0.59, and AGP in WRA = 0.53; apply adjustments only to individuals with either CRP > CRP_ref_, AGP > AGP_ref_, or both.

1 sTfR was adjusted for AGP but not for CRP per a biological rationale as described elsewhere (10). AGP, α1-acid glycoprotein; BRINDA, Biomarkers Reflecting Inflammation and Nutritional Determinants of Anemia; CF, correction factor; CRP, C-reactive protein; GM, geometric mean; MB, micronutrient biomarker; PSC, preschool children; RC, regression correction; ref, reference value; sTfR, soluble transferrin receptor; WRA, women of reproductive age; β_1_, CRP regression coefficient; β2, AGP regression coefficient.

2 Inflammation status groups—CRP: *1*) no inflammation (CRP ≤5 mg/L) (ref); *2*) inflammation (CRP >5 mg/L). AGP: *1*) no inflammation (AGP ≤1 g/L) (ref); *2*) inflammation (AGP >1 g/L). CRP and AGP: *1*) no inflammation (CRP ≤5 mg/L and AGP ≤1 g/L) (ref); *2*) incubation (CRP >5 mg/L and AGP ≤1 g/L); *3*) early convalescence (CRP >5 mg/L and AGP >1 g/L); *4*) late convalescence (CRP ≤5 mg/L and AGP >1 g/L).

### Prevalence of inflammation in women and children

**FIGURE 1:**
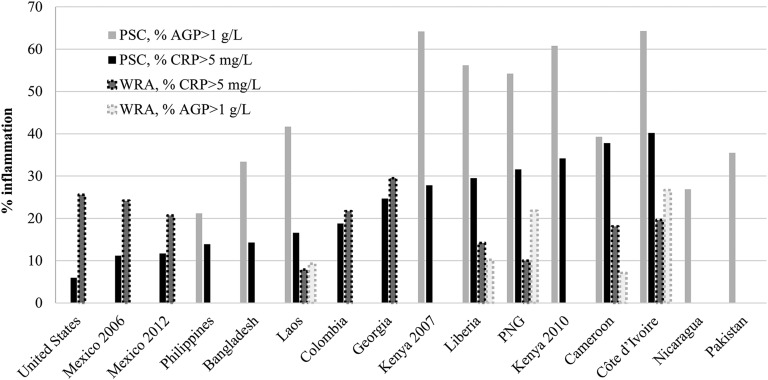
Prevalence of inflammation in PSC and WRA: the BRINDA project. Countries are ordered from lowest to highest inflammation on the basis of elevated CRP in PSC. AGP, α1-acid glycoprotein; BRINDA, Biomarkers Reflecting Inflammation and Nutrition Determinants of Anemia; CRP, C-reactive protein; PNG, Papua New Guinea; PSC, preschool children; WRA, nonpregnant women of reproductive age. Figured created from data presented in Merrill et al. (28) with permission.

The total sample size for the inflammation analysis was 29,765 PSC and 25,731 WRA (28). A total of 1135 PSC and 3196 WRA were included from the NHANES data set. The prevalence of inflammation (elevated CRP or AGP) in PSC varied from 6.0% (United States) to 64.3% (Cote d’Ivoire) (**Figure 1**). In countries that measured both CRP and AGP concentrations, inflammation in PSC was considerably more common when defined by elevated AGP (median: 54.2%) than by elevated CRP (median: 29.5%). However, for WRA, the prevalence of inflammation was similar as defined by AGP and CRP concentrations (medians: 15.6% for AGP and 14.1% for CRP).

The overall prevalence of inflammation (elevated CRP or AGP) appeared to be lower in WRA (median: 20.0%) than in PSC (median: 57.0%). When the data from just low– and medium–infection-burden countries (United States, Republic of Georgia, Mexico, and Colombia) were considered, the prevalence of an elevated CRP concentration in WRA (median: 24.3%) appeared to be higher than that in PSC (median: 11.7%). For example, the prevalence of inflammation in WRA in the United States was 4 times higher than that in PSC (25.6% compared with 6.0%). Conversely, in the high–infection-burden countries, the prevalence of an elevated CRP concentration in WRA (median: 17.8%) was lower than that in PSC (median: 37.5%) in data sets that included information on both populations.

### Relation between inflammation and iron indicators

**FIGURE 2:**
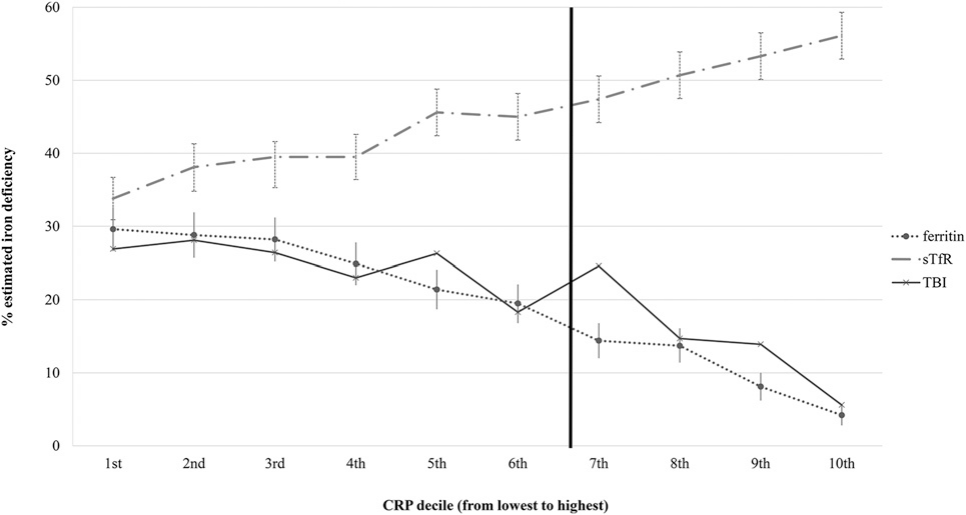
Pooled estimated (95% CI) ID in preschool children according to ferritin and sTfR concentrations and TBI by CRP decile: the BRINDA project. The analysis was restricted to countries that measured both CRP and α1-acid glycoprotein; cutoffs to define estimated ID were as follows: ferritin concentration <12 μg/L, sTfR concentration >8.3, and TBI <0. The bold vertical line indicates the commonly used cutoff for CRP. BRINDA, Biomarkers Reflecting Inflammation and Nutrition Determinants of Anemia; CRP, C-reactive protein; ID, iron deficiency; sTfR, soluble transferrin receptor; TBI, total body iron. Adapted from Namaste et al. (30) with permission.

The total sample size for the SF analysis was 27,865 PSC and 24,844 WRA (26); the sample size for sTfR was 11,913 PSC and 11,173 WRA (27); and the sample size for TBI was 8413 PSC and 4258 WRA (25). With the use of pooled data of countries that measured both CRP and AGP concentrations, the relation between the estimated prevalence of ID according to each of the 3 iron status indicators and CRP deciles appeared to follow a linear pattern in PSC (**Figure 2**). The estimates of ID varied between CRP deciles even at low concentrations of CRP (e.g., those less than the cutoff of 5 mg/L). For example, the overall unadjusted prevalence of ID in PSC on the basis of a low SF concentration was 19.5% but varied from 4.2% at the highest (10th) CRP decile to 29.6% at the lowest (first) CRP decile. The prevalence of an elevated sTfR concentration varied from 56.1% to 33.8% at the 10th and first CRP deciles, respectively. On the basis of low TBI, the prevalence of estimated ID varied from 3.6% to 29.4% at the 10th and third CRP deciles, respectively. TBI at the first and second CRP deciles were lower than expected, which were likely due to the small sample size. The relations in PSC between estimated ID and AGP deciles were similar (30). In WRA, there was also a similar pattern between the estimated prevalence of ID and inflammation decile; however, the slope was not as steep (30).

**FIGURE 3:**
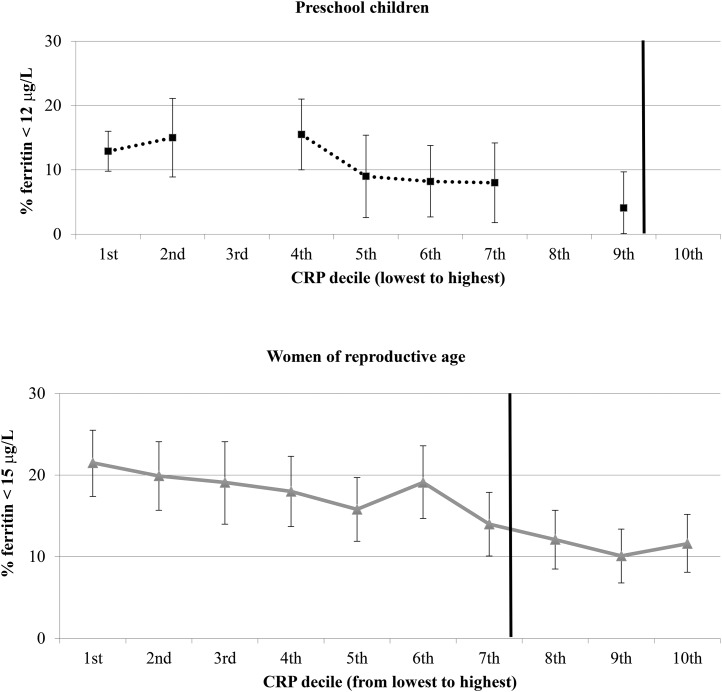
Estimated (95% CI) ID according to low ferritin concentrations by CRP decile in NHANES preschool children and nonpregnant women of reproductive age. The bold vertical line indicates the commonly used cutoff for CRP. The small sample size in preschool children led to missing values at CRP deciles 3, 8, and 10. CRP, C-reactive protein; ID, iron deficiency.

In the United States, the distributions of CRP concentrations in both PSC and WRA were left skewed with most values at 0.1 mg/L (data not shown), and AGP concentrations were not available. Thus, the estimated prevalence of ID for both PSC and WRA on the basis of a low SF concentration by CRP decile did not vary as much as in the pooled BRINDA analysis (**Figure 3**). In addition, the small sample size led to missing values at some CRP deciles. Nevertheless, the proportion of PSC with low SF concentrations ranged from ∼4% at the highest CRP decile to 13% at the lowest CRP decile. The effect of CRP on SF concentrations was more pronounced in WRA in the United States with an estimated ID ranging from 11% to 22% in the highest and lowest CRP deciles, respectively.

### Effects of CRP and AGP adjustment on estimated ID

**Table 2** summarizes the median change in estimated ID by adjustment approach in both PSC and WRA for the pooled analysis of countries that measured CRP and AGP. Overall, all adjustment approaches increased the estimated prevalence of ID when SF concentrations were used, decreased the estimated prevalence of ID when sTfR concentrations were used, and increased the estimated prevalence of ID when TBI was used. The regression approach produced the largest difference in estimated ID by increasing the prevalence of low SF concentrations by 25 percentage points (pps) and increasing the prevalence of low TBI by 14 pps in PSC. In contrast, the prevalence of an elevated sTfR concentration decreased by 15 pps in PSC. The overall effects of the exclusion and correction-factor approaches were similar. The pattern was similar in WRA except that the magnitude of effect was smaller for each correction approach (Table 2).

**TABLE 2:** Summary of changes in estimated ID by adjustment method with the use of pooled data in preschool children and nonpregnant women of reproductive age: the BRINDA project^1^

Approach	Percentage point difference		
	SF^2^	sTfR	TBI
Preschool children			
Sample size (surveys), *n*	8413 (8)	9281 (9)	8413 (8)
Exclusion	+6.5 (2.6–15.4)^3^	−6.5 (0.4–14.2)^4^	+4.8 (1.7–12.9)^3^
Correction factor	+6.5 (2.6–16.6)	−6.0 (0.7–12.4)	+3.6 (1.5–13.4)
Regression correction	+24.7 (8.1–35.6)	−14.6 (0.8–23.3)	+14.4 (4.1–25.9)
Nonpregnant women of reproductive age			
Sample size (surveys), *n*	4258 (4)	5004 (5)	4258 (4)
Exclusion	+1.6 (1.1–3.0)^3^	−1.9 (0.9–2.5)^4^	+1.1 (1.0–2.7)^3^
Correction factor	+1.6 (1.2–2.6)	−1.6 (0.9–2.7)	+1.0 (0.2–1.4)
Regression correction	+7.5 (4.1–10.7)	−9.5 (1.9–13.8)	+2.7 (0.9–5.6)

1 Values are absolute medians (ranges), unless otherwise indicated. On the basis of data sets that had both CRP and AGP compared with no adjustment. Data were derived from Namaste et al. (26), Rohner et al. (27), and Mei et al. (25) with permission. AGP, α1-acid glycoprotein; BRINDA, Biomarkers Reflecting Inflammation and Nutrition Determinants of Anemia; CRP, C-reactive protein; ID, iron deficiency; SF, serum ferritin; sTfR, soluble transferrin receptor; TBI, total body iron.

2 Note that plasma ferritin was used in some surveys.

3 Excluded on the basis of a CRP concentration >5 mg/L or AGP concentration >1 g/L.

4 Excluded on the basis of an AGP concentration >1g/L.

After dividing countries by infection burden, we were able to apply inflammation adjustments only for CRP and SF concentrations because of the lack of AGP data in low– and medium–infection-burden countries (Colombia, Mexico, and the United States). Compared with unadjusted estimates, there was a median 3.6-pp increase in estimated ID (SF concentration <12 μg/L) in PSC in low– and medium–infection-burden countries with the use of the regression approach compared with a 14.3-pp increase in high– and very high–infection-burden countries (data not shown). In WRA, there were no differences in adjustment by infection burden (6.9-pp increase in low– and medium–infection-burden countries compared with a 6.0-pp increase in high– and very high–infection-burden countries). In the NHANES, there was a 2.8-pp increase in estimated ID with the use of low SF in PSC and a 7.0-pp increase in WRA with adjustment for CRP alone (data not shown). Adjustments were not made for sTfR concentrations or TBI in the United States because of the lack of AGP data (25, 27).

## DISCUSSION AND RESEARCH GAPS

With the use of data from the BRINDA project, we showed that all of the examined indicators of iron status (SF and sTfR concentrations and TBI) were affected by inflammation even in low–infection-burden countries including the United States. In addition, iron-status indicators changed at low concentrations of CRP and AGP, thereby suggesting that continuous correction using a regression-correction approach may better account for the full range and severity of inflammation than would the exclusion or correction-factor approaches that rely on dichotomous cutoffs to define inflammation. The overall prevalence of estimated ID as defined by low SF concentrations was higher by ∼25 pps in PSC and by 8 pps in WRA when adjusting for both CRP and AGP. In WRA in the United States, which is a low–infection-burden country, ID was 7 pps higher when adjusting for CRP alone.

A major strength of the BRINDA analysis is that it included a large sample size from numerous high-quality, population-based surveys across the globe. However, the data were also heterogeneous, and some countries had gaps in biomarker data, which limited some comparisons across countries. Limitations in data availability may have also resulted in the regression-correction equation not being entirely the same in each country; with more data, it may be possible to group countries on the basis of infection burden, e.g., and apply a common inflammation regression correction. In addition, causality inferences are limited on the basis of the cross-sectional design of all of the surveys. It remains uncertain whether the regression approach can be applied to improve the detection of iron status in the prospectively evaluated clinical context. Finally, the validity of the regression-correction approach to adjust estimates of ID could not be evaluated because none of the surveys had a gold-standard indicator of ID.

The BRINDA findings highlight the need to measure biomarkers of inflammation when assessing iron status at the population level. Recent studies in infants and PSC have suggested that predictors of inflammation vary by setting, and sociodemographic or morbidity information alone cannot reliably identify inflammation, thus suggesting the importance of measuring biochemical indicators of subclinical inflammation (28, 34). Both CRP and AGP concentrations may be important because they reflect different phases of the APR that range from acute infection (e.g., rapid onset within 1 h) to chronic inflammation (e.g., rising after 24 h and lasting >4–5 d) (9). The development of the fixed correction-factor approach by Thurnham et al. (35) was instrumental in recommending the assessment of both CRP and AGP to divide apparently healthy populations in a reference group and 3 inflammation groups on the basis of concentrations of these 2 AP proteins. As countries continue to develop and face increases in noncommunicable disease burdens such as obesity, the need to assess chronic inflammation becomes even more important (8). The inclusion of a biomarker of chronic inflammation such as AGP is particularly needed in future NHANES and nutrition surveys in other low–infection-burden countries to assess the impact of chronic inflammation on estimated ID when the BRINDA approach is used.

Key research gaps for assessing iron status in settings of inflammation are summarized in **Table 3**. Rapid, field-friendly, and affordable tests for the assessment of both inflammatory and iron-status indicators are needed for use at the individual and population levels globally. The standardization of these tests will assist in the comparability of results over time and across populations. In addition, having a better understanding of the etiology of elevated markers of inflammation in different population groups will help determine whether the relation between biomarkers of inflammation and iron status is consistent or differs by the cause of inflammation (e.g., infection, cancer, autoimmune disease, or obesity). Liver disease, in particular, may directly elevate SF concentrations independent of inflammation through hepatocyte damage and leakage of intracellular contents. Obesity is associated with inflammation as well as hepatic damage because of nonalcoholic steatohepatitis (36), and hence, SF concentrations may be elevated in this population even when inflammatory biomarkers are not. Therefore, in populations in developed countries (and those making the transition), it may also be pertinent to define whether adjustments for SF concentrations with the use of biomarkers of liver disease (or other clinical factors) are relevant and feasible. Indeed, there is evidence that SF concentrations are increasing in these populations along with BMI (37), and as such, a combination of biomarkers may be needed to assess iron status in these populations. The potential limitations of SF concentrations underscores the need for further studies to define the role of other iron indicators especially those that respond to iron interventions and, thus, can be used to monitor the impact of public health programs. Finally, carefully designed studies of iron status that compare commonly used indicators of iron status such as the SF concentration against the gold standard of bone marrow iron stores that also include biomarkers of inflammation are needed to validate the different approaches of adjusting iron status for inflammation.

**TABLE 3:** Key research gaps for the assessment of iron status in settings of inflammation^1^

Problem or question	Studies or technological developments needed (examples)
Need field-friendly and cost-effective inflammatory biomarkers that are standardized across laboratories.	Simple, accurate, reliable, and inexpensive inflammation biomarkers tests
Do the characteristic patterns of change in AP proteins differ according to population group and inflammation etiologies (e.g., infection, obesity, and trauma)?	Ecologic studies on inflammation
What is the magnitude and duration of the effects of inflammation on iron status and iron indicators? How long after the inflammatory event do the iron indicators become useful indexes?	Longitudinal studies in children and adults that define which and when commonly used iron-status indicators are affected by inflammation including infectious and noninfectious triggers of inflammation
How responsive are iron indicators to interventions, and which are more useful to monitor trends and the impact of public health interventions (e.g., bioindicators)?	Effectiveness studies unadjusted and adjusted for inflammation
How close is the inflammation-adjusted prevalence to the true prevalence of ID?	Validation studies that measure indicators of iron status, inflammation, and gold-standard bone marrow iron

1 AP, acute phase; ID, iron deficiency.

In summary, both acute and chronic inflammation as measured by CRP and AGP concentrations alter commonly measured indicators of iron status (SF and sTfR concentrations and TBI) and the regression-correction approach is one method to ameliorate inflammation-confounded estimates of population-level ID. In clinical settings, the application of inflammation-correction approaches is a burgeoning research area that will help elucidate the mechanisms underlying the effects of inflammation on iron metabolism.
